# Supplementary material for: Unravelling Regioselectivity of Leuconostoc citreum ABK-1 Alternansucrase by Acceptor Site Engineering
Source: Int J Mol Sci. 2021 Mar 22;22(6):3229. doi: 10.3390/ijms22063229 (PMC8005217; doi:10.3390/ijms22063229)
Supplement: Supplementary file 1 [file ijms-22-03229-s001.pdf]

Unravelling regioselectivity of *Leuconostoc citreum* ABK-1 alternansucrase by acceptor site engineering

Karan Wangpaiboon [1], Thassanai Sitthiyotha[2], Surasak Chunsrivirot[1, 2], Thanapon Charoenwongpaiboon[3], and Rath Pichyangkura [1, *]

[1] Department of Biochemistry, Faculty of Science, Chulalongkorn University, Bangkok 10330, Thailand
[2] Structural and Computational Biology Research Unit, Department of Biochemistry, Faculty of Science, Chulalongkorn University, Pathumwan, Bangkok 10330, Thailand
[3] Department of Chemistry, Faculty of Science, Silpakorn University, Nakhon Pathom 73000, Thailand
* Correspondence: Rath Pichyangkura, E-mail: prath@chula.ac.th

Table 1. List of primers for site-directed mutagenesis at W675 position.

primer name	primer sequence (5'-3')	remark
F_altNcoI	GGGAGAGTAATCCATGGAACAACAAG	Forward primer
R_Del7	CAGCCGGATCCTCGAGTTAAGCTTGC	Revered primer
F_W675Y	CTATTTTAGAAGACTATAATGGCAAAGATCCTC	mutation at W675 to Y
R_W675Y	GAGGATCTTTGCCATTATAGTCTTCTAAAATAG	mutation at W675 to Y
F_W675F	CTATTTTAGAAGACTTTAATGGCAAAGATCCTC	mutation at W675 to F
R_W675F	GAGGATCTTTGCCATTAAAGTCTTCTAAAATAG	mutation at W675 to F
F_W675L	CTATTTTAGAAGACCTGAATGGCAAAGATCCTC	mutation at W675 to L
R_W675L	GAGGATCTTTGCCATTCAGGTCTTCTAAAATAG	mutation at W675 to L
F_W675I	CTATTTTAGAAGACATTAATGGCAAAGATCCTC	mutation at W675 to I
R_W675I	GAGGATCTTTGCCATTAATGTCTTCTAAAATAG	mutation at W675 to I
F_W675S	CTATTTTAGAAGACTCCAATGGCAAAGATCCTC	mutation at W675 to S
R_W675S	GAGGATCTTTGCCATTGGAGTCTTCTAAAATAG	mutation at W675 to S
F_W675H	CTATTTTAGAAGACCATAATGGCAAAGATCCTC	mutation at W675 to H
R_W675H	GAGGATCTTTGCCATTATGGTCTTCTAAAATAG	mutation at W675 to H
F_W675N	CTATTTTAGAAGACAACAATGGCAAAGATCCTC	mutation at W675 to N
R_W675N	GAGGATCTTTGCCATTGTTGTCTTCTAAAATAG	mutation at W675 to N
F_W675D	CTATTTTAGAAGACGATAATGGCAAAGATCCTC	mutation at W675 to D
R_W675D	GAGGATCTTTGCCATTATCGTCTTCTAAAATAG	mutation at W675 to D

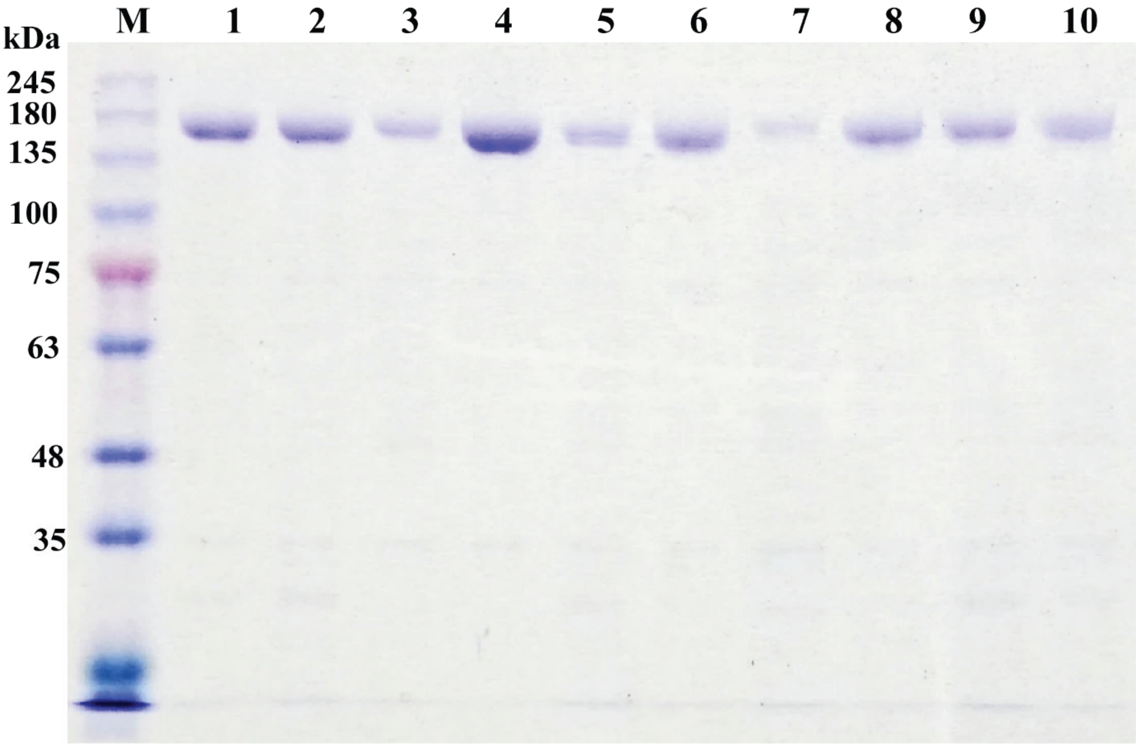

Figure S1. SDS-PAGE analysis of purified alternansucrase and variants. Lane M is protein molecular weight marker. Lane1 is WT ALT. Lane2-10 are W675Y, W675F, W675H, W675N, W675D, W675S, W675I, W675L, and W675A, respectively.

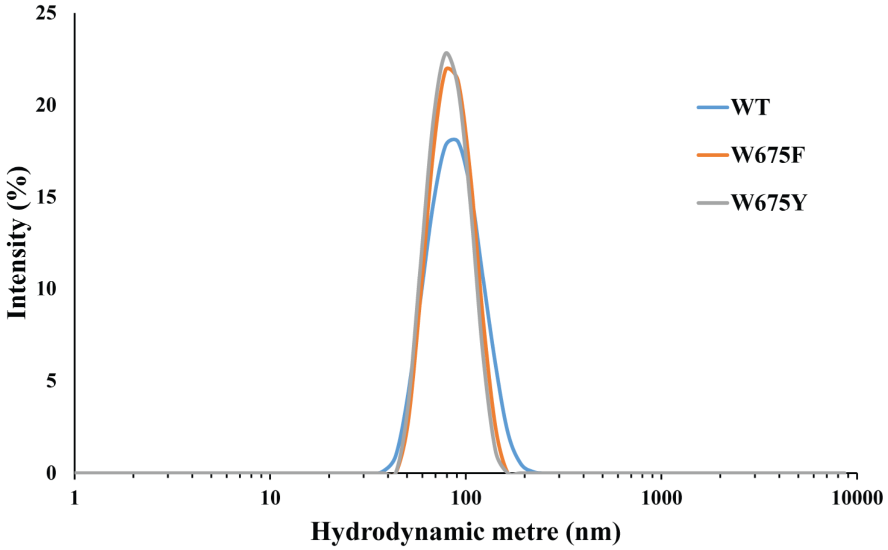

Figure S2. Hydrodynamic size of alternan-nanoparticles. The purified polymer of WT, W675F, and W675Y were ananlysed by Malvern Nanosizer ZS at 25 °C.

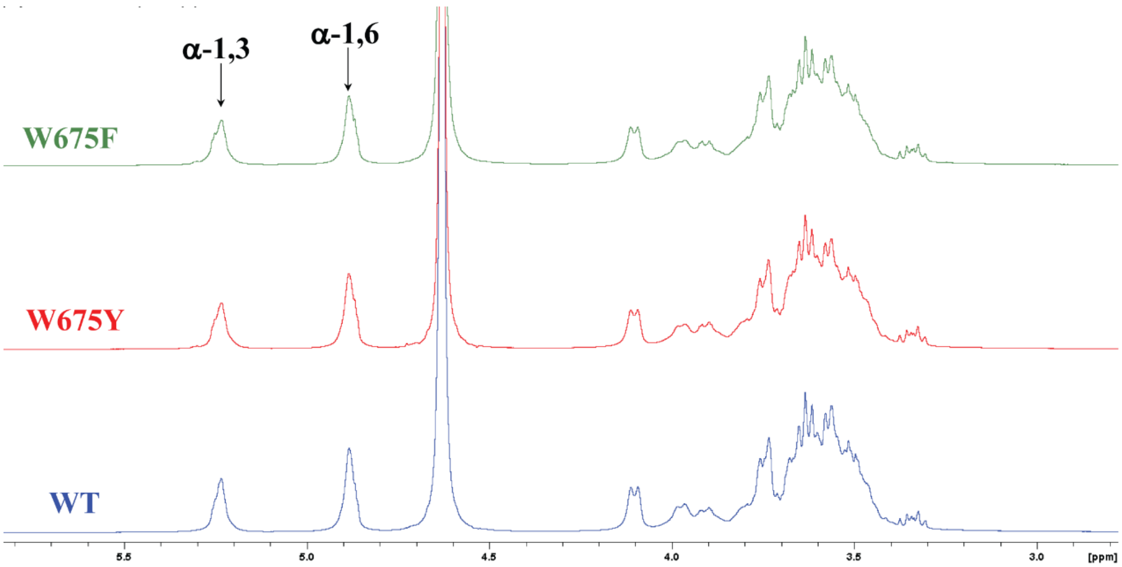

Figure S3. ^{1}H NMR spectra of glucan polymers. The purified glucans polymer of WT, W675Y, and W675F were recorded at 500 MHz in D_2O at 298 K.

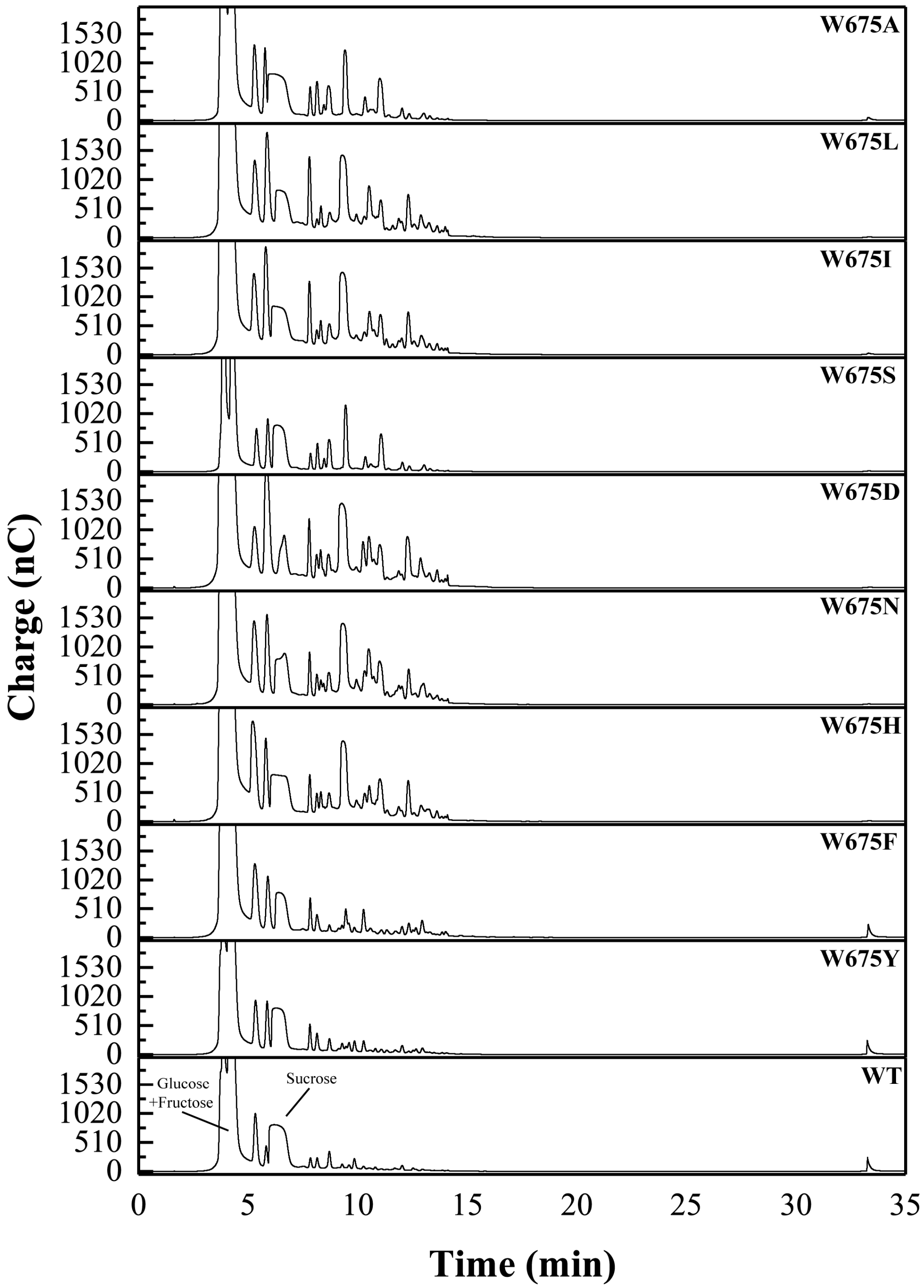

Figure S4. HPAEC-PAD analysis of sucrose reaction. The oligosaccharide patterns of sucrose reactions produced from WT and variants were analysed by HPAEC-PAD. The reactions comprised 200 mM sucrose, 50 mM sodium citrate buffer pH 4.0 and 0.1 U/mL enzyme. The reactions were incubated at 37 °C for 20 hr.

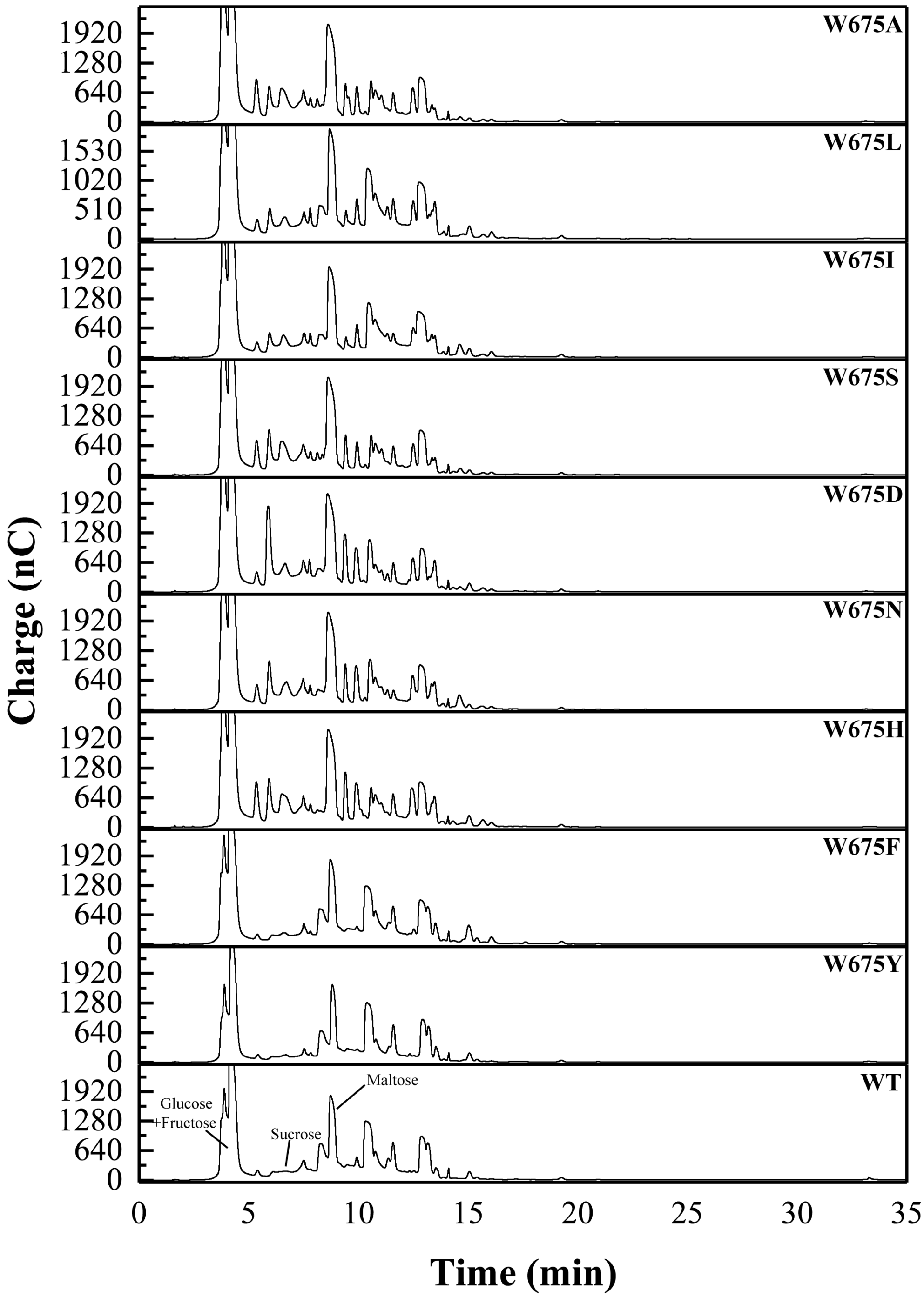

Figure S5. HPAEC-PAD analysis of maltose-acceptor reaction. The oligosaccharide patterns of maltose-acceptor reactions produced from WT and variants were analysed by HPAEC-PAD. The reactions were conducted in 100 mM maltose, 100 mM sucrose and 0.1 U/mL enzymes in 50 mM sodium citrate buffer pH 4.0. Then, the reactions were incubated at 37 °C for 20 hr.

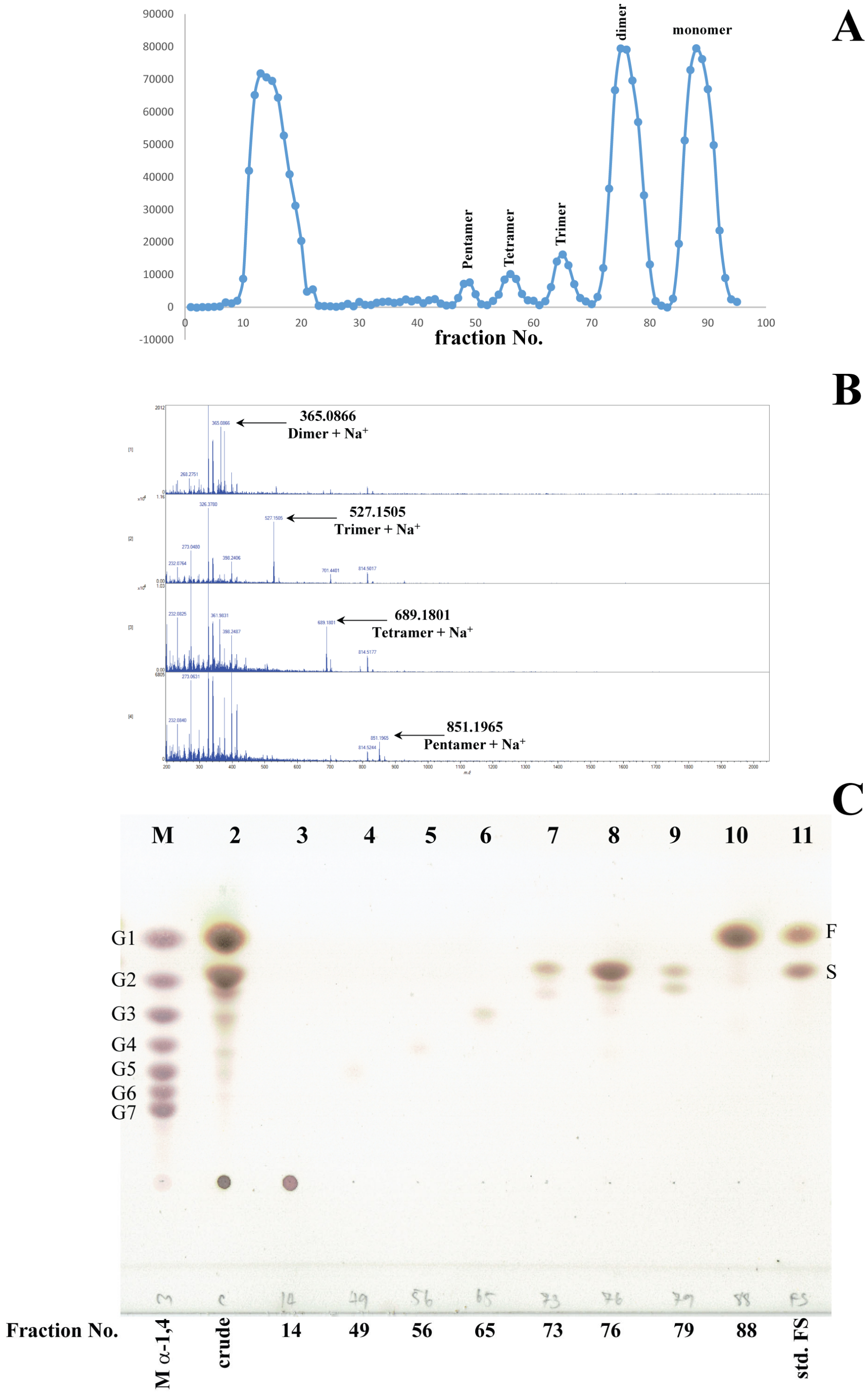

Figure S6. Purification of products produced from WT reaction. The reactions comprised 200 mM sucrose, 50 mM citrate buffer pH 4.0 and 0.1 U/mL enzyme. The reactions were incubated at 37 °*C* for 20 hr. Crud product approximately 300 mg were purified by Bio-Gel P-2 (Bio-Rad) column and then analysed by MALDI-TOF MS and TLC, respectively.

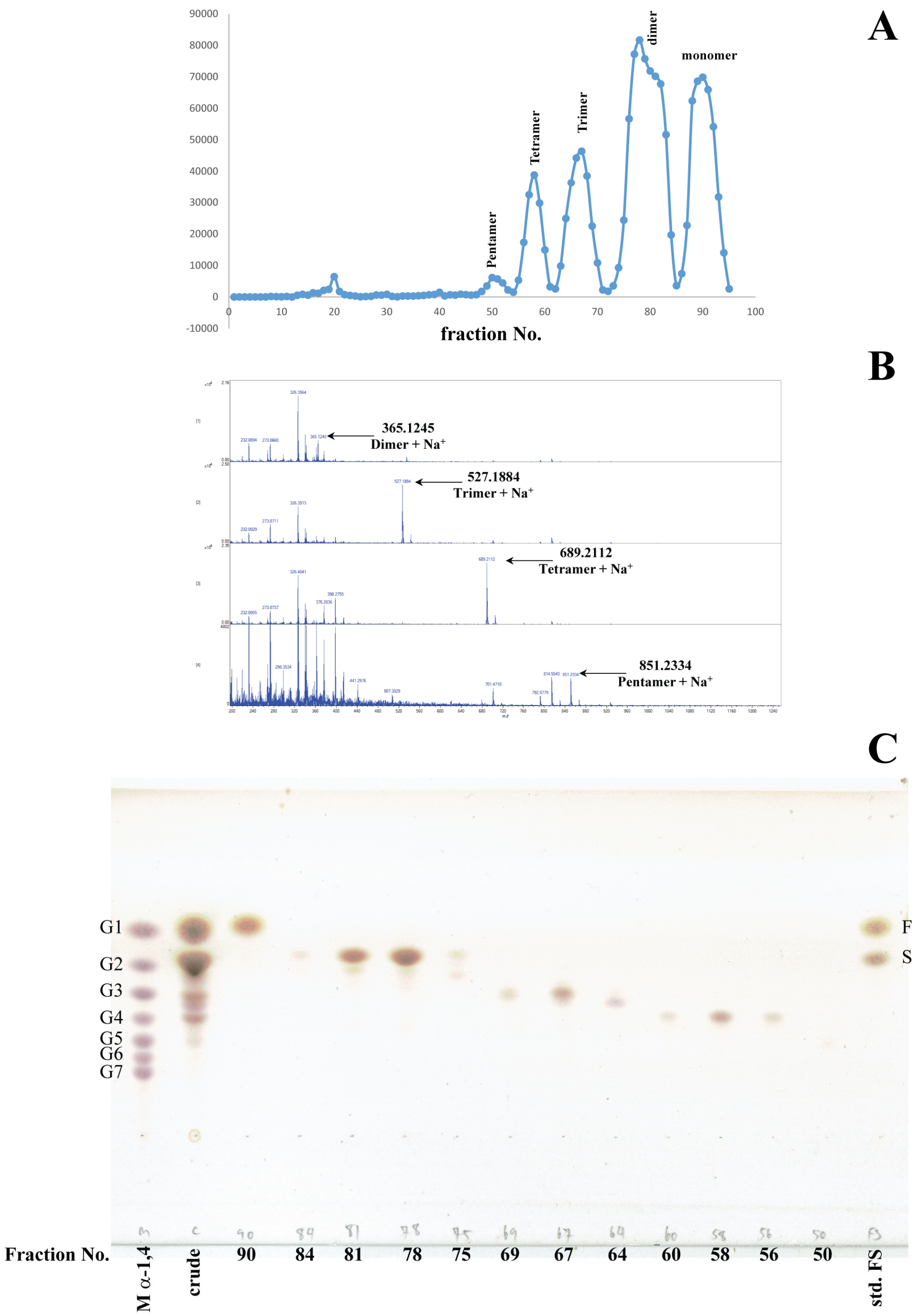

Figure S7. Purification of products produced from W675A reaction. The reactions comprised 200 mM sucrose, 50 mM citrate buffer pH 4.0 and 0.1 U/mL enzyme. The reactions were incubated at 37 °*C* for 20 hr. Crud product approximately 300 mg were purified by Bio-Gel P-2 (Bio-Rad) column and then analysed by MALDI-TOF MS and TLC, respectively.

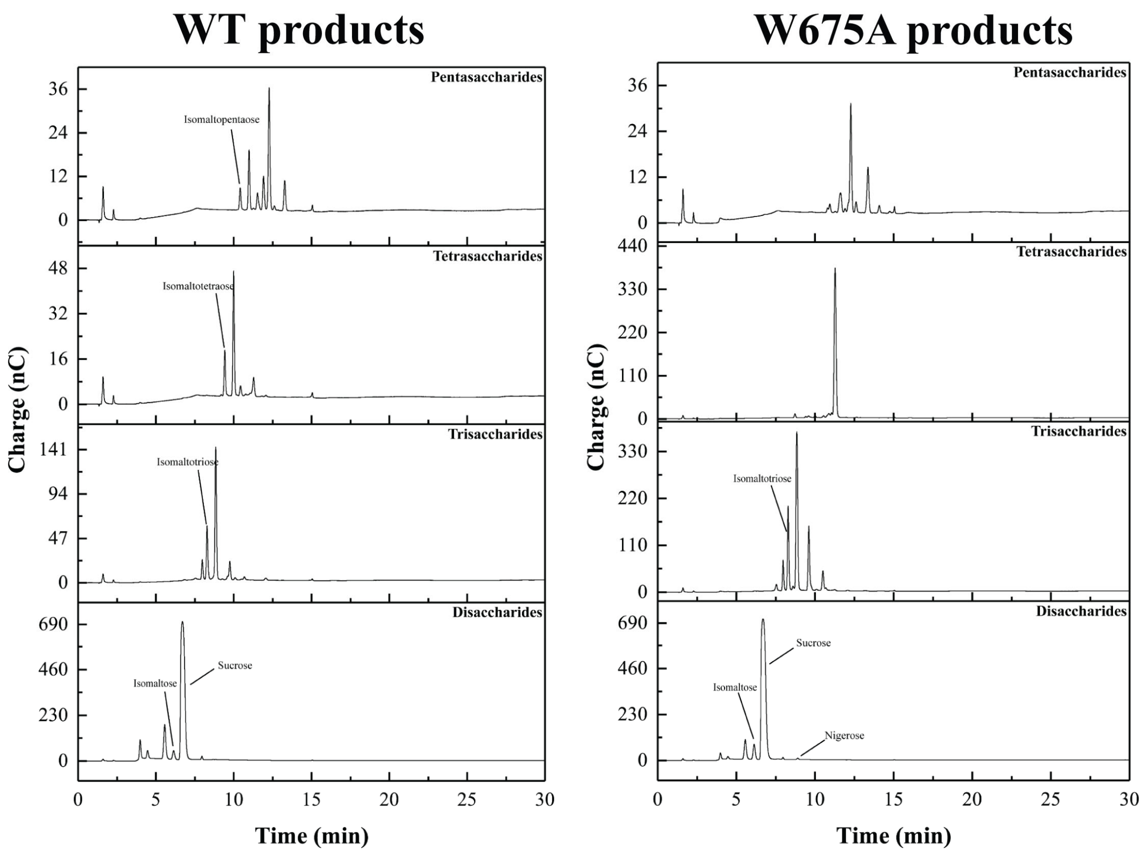

Figure S8. HPAEC-PAD profiles products produced from WT and W675A reaction via sucrose reaction.

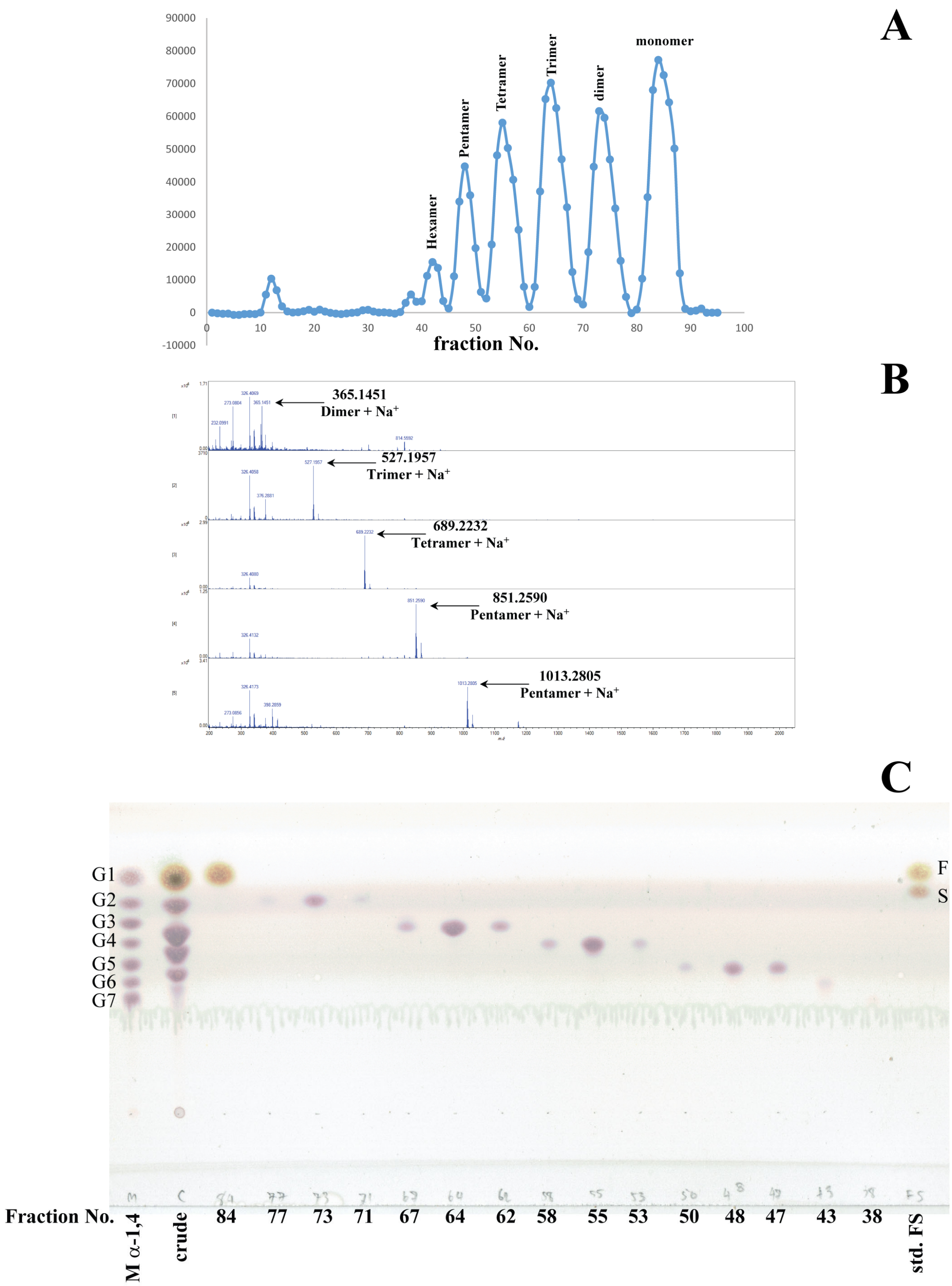

Figure S9. Purification of maltose reaction from WT reaction. The reactions comprised 100 mM sucrose, 100 mM maltose50 mM citrate buffer pH 4.0 and 0.1 U/mL enzyme. The reactions were incubated at 37 °*C* for 20 hr. Crud product approximately 350 mg were purified by Bio-Gel P-2 (Bio-Rad) column and then analysed by MALDI-TOF MS and TLC, respectively.

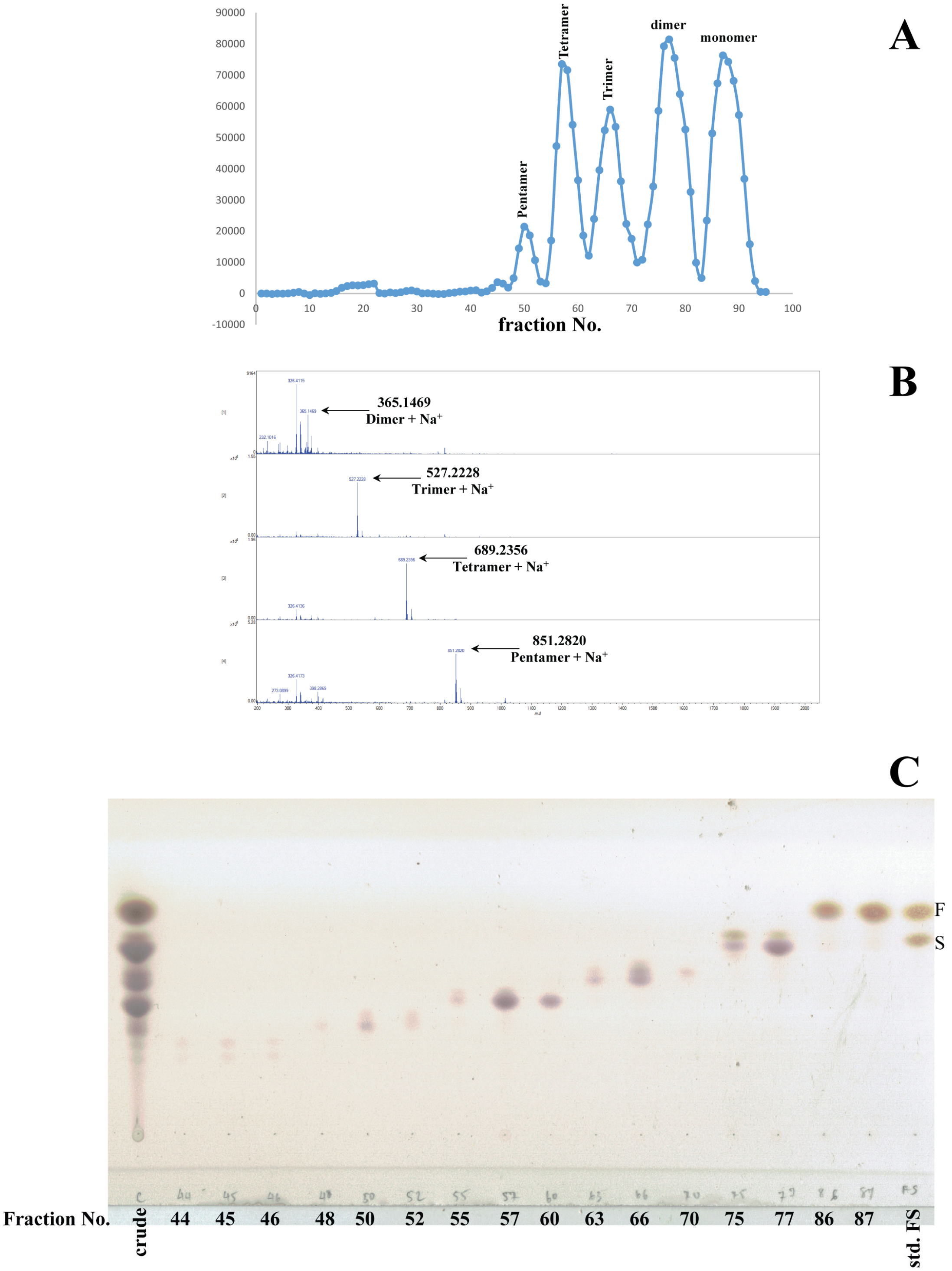

Figure S10. Purification of maltose reaction from WT reaction. The reactions comprised 100 mM sucrose, 100 mM maltose50 mM citrate buffer pH 4.0 and 0.1 U/mL enzyme. The reactions were incubated at 37 °*C* for 20 hr. Crud product approximately 350 mg were purified by Bio-Gel P-2 (Bio-Rad) column and then analysed by MALDI-TOF MS and TLC, respectively.

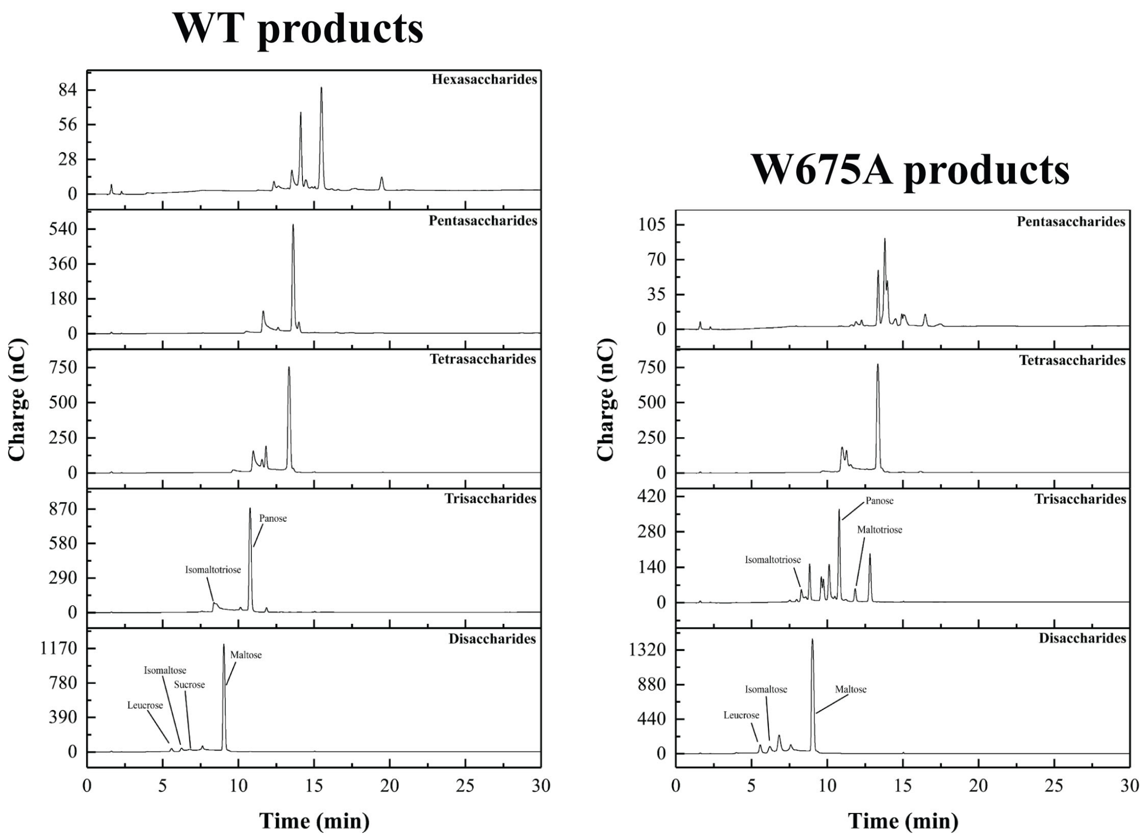

Figure S11 HPAEC-PAD profiles of maltose reaction produced from WT and W675A

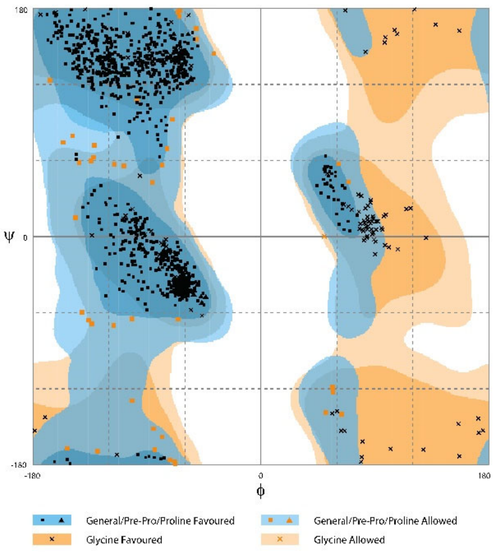

Figure S12: Ramachandran plot of the homology model of *Leuconostoc citreum* ABK-1 alternansucrase.

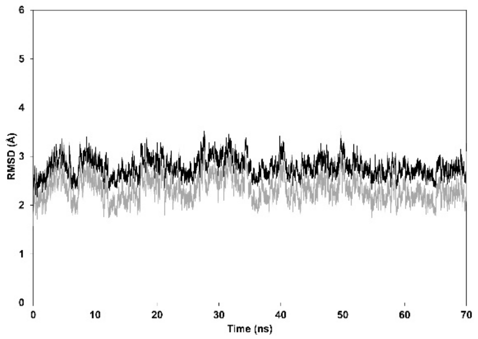

Fig S13: RMSD plots of alternansucrase with glc-D635 intermediate. The RMSD values of all atoms and backbone atoms are shown in black and grey, respectively.

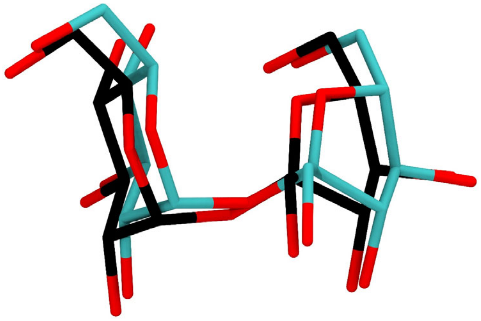

Fig S14: Superimposition of the crystal binding conformation (black) and the best docked conformation (cyan).

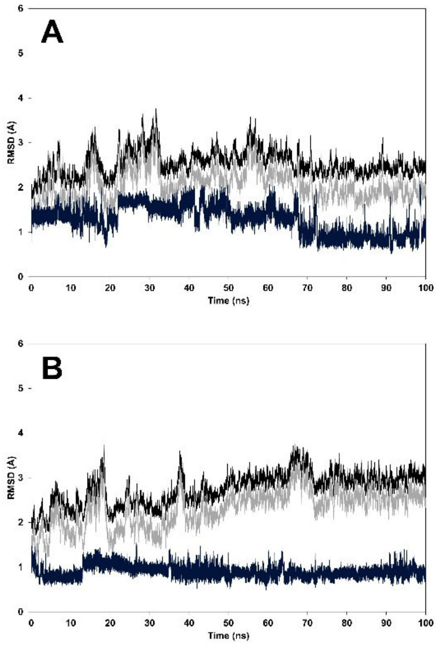

Fig S15: RMSD plots of A) maltose-ALT$_{wt}$ and B) maltose-ALT$_{W675A}$ complexes. The RMSD values of all atoms, backbone atoms and ligand atoms are shown in black, grey and blue, respectively.

Supplementary Information

Methods

Molecular dynamics simulations

The structure of maltose was obtained from the crystal structure of *Lactobacillus reuteri* glucansucrase (PDB: 3KLL [1]). LEaP module in AMBER18 [2] with GLYCAM06j-1 force field parameters [13] was used to build structure of maltose. To reduce the unfavorable interactions, this structure was immersed in an isomeric truncated octahedral box of TIP3P water molecules, and minimized using 2,500 steps of steepest descent and 2,500 steps of conjugate gradient. SWISS-MODEL server [3-6] was used to construct the homology model of *Leuconostoc citreum* ABK-1 alternansucrase based on the crystal structure of *Leuconostoc mesenteroides* Alternansucrase (PDB: 6HVG [7]), which has the highest sequence identity to the target sequence (97.40%). RAMPAGE server [14] was employed to construct Ramachandran plot to evaluate the quality of the constructed homology model of *Leuconostoc citreum* ABK-1 alternansucrase as shown in S12 Fig. The majority of amino acid residues are in favored region (96.6%) and allowed region (5.6%), indicating reasonable qualities of this homology model. Moreover, the catalytic residues of this homology model (D635, E673, and D767) were found in similar positions to those of *Leuconostoc mesenteroides* alternansucrase, where they should be able to catalyze the reaction. H^{++} server [8] was used to protonate all ionized amino acids at the experimental pH 4.0. To construct the structure of glucosyl-D635 (glc-D635) intermediate, the structure of glc-D635 intermediate was obtained from the crystal structure of *Neisseria polysaccharea* amylosucrase (PDB: 1S46 [9]). Gaussian09 program [10] with HF/6-31G* basis set was used to calculate the atomic charges and the electrostatic potential (ESP) charges of the glc-D635 intermediate. Antechamber module in AMBER18 was employed to fit the ESP charge of the glc-D635 intermediate into restrained ESP (RESP) charge, and other force filed parameters were generated from general AMBER force field (GAFF). LEaP module was used to construct the structure of alternansucrase with the glc-D635 intermediate, using ff14SB parameters. The structure of alternansucrase with the glc-D635 intermediate was immersed in an isomeric truncated octahedral box of TIP3P water molecules, using the buffer distance of 13 Å. Sodium ions (Na^+) were added to neutralize the system. This system was minimized to remove the unfavorable interaction using the five step procedure, all steps used 5,000 steps of steepest descent and 5,000 steps of conjugate gradient with different restraints on the proteins. At the beginning, the heavy atoms of protein were restrained with a force constant of 10 kcal/ (mol Å^2), while the hydrogen atoms and water molecules were minimized. The backbone of the protein was then restrained with force constants of 10, 5 and 1 kcal/ (mol Å^2), respectively. The entire system was finally minimized without any restraining force.

The system was subsequently simulated with the periodic boundary condition using the GPU (CUDA) version of PMEMD module [15-17]. The SHAKE algorithm [18] was employed to constrain all bonds involving hydrogen atoms, allowing 0.002 ps time step simulations. The Langevin dynamics [19] was used to control the temperature with a collision frequency of 1.0 ps^{-1}. The system was simulated in the NVT ensemble to heat from 0 K to the experimental temperature of 310 K for 200 ps, while the backbone of protein was restrained with a force constant of 10 kcal/ (mol Å^2). Then, the system was subsequently equilibrated for 300 ps in the NVT ensemble with no restraining force. The system was further simulated for 70 ns in the

NPT ensemble at 310 K and 1 atm. To analyze the structural stability of the system, Root Mean Square Deviation (RMSD) values were calculated (S13 Fig). The last 20 ns trajectories with stable RMSD values were selected for clustering by MMTSB tool set [11] based on their structural similarities as analyzed by RMSD values of heavy atoms. The structure that is the most similar to the average structure was selected to be a centroid, a representative structure, for further analyses.

The crystal structure of sucrose was redocked into the active site of the crystal structure of *Lactobacillus reuteri* glucansucrase (PDB: 3HZ3 [1]) to determine whether Vina-Carb program [12] and its parameters were reasonable for the studied system. The best docked and crystal binding conformations were similar with the RMSD value of 0.54 Å (S14 Fig), indicating that Vina-Carb and its parameters were reasonably appropriate for our system. To predict the catalytically competent binding conformations of maltose in the binding site of *Leuconostoc citreum* ABK-1 alternansucrase, the constructed structure of maltose was docked into the active site of the representative structure of alternansucrase with the glc-D635 intermediate to construct maltose-ALT$_{wt}$ complex, using a grid box of 30 Å x 30 Å x 30 Å with a grid spacing of 1 Å. 20 independent docking runs were performed, resulting in the total of 2,000 binding conformations. The catalytically competent binding conformation of maltose in the active site of alternansucrase was selected if the distance from O6 hydroxyl group of the non-reducing terminal glucose of maltose to C1 atom of the glc-D635 intermediate was less than or equal to 3.5 Å [7]. To identify the most reasonable catalytically competent binding conformation, 54 binding conformations that passed the distance criterion were superimposed with the crystal structure of maltose in the acceptor binding site of *Lactobacillus reuteri* glucansucrase (PDB: 3KLL); the binding conformation with the lowest heavy-atom RMSD values was selected. To construct the structure of W675A complex, W675 of maltose-ALT$_{wt}$ complex was mutated to A675 to construct the maltose-ALT$_{W675A}$ complex. The maltose-ALT$_{wt}$ and maltose-ALT$_{W675A}$ complexes were then immersed in an isomeric truncated octahedral box of TIP3P water molecules with a buffer distance of 13 Å and neutralized by sodium ions (Na^+). All systems were minimized, heated, equilibrated and simulated for 100 ns of production runs with similar setup procedure.

RMSD values were computed to monitor the structural stability during the simulations of all systems. The proximity between atoms necessary for transglycosylation was measured by the distance between O2, O3, O4, O6 hydroxyl groups of the non-reducing terminal glucose of maltose and C1 atom of the glc-D635 intermediate. Hydrogen bond interactions were calculated based on hydrogen bond occupations between binding residues and maltose. Hydrogen bond was determined to occur if the following criteria were met: (i) a proton donor-acceptor distance ≤ 3.5 Å and (ii) a donor-H-acceptor bond angle ≥ 120°.

References

1. Vujičić-Žagar A, Pijning T, Kralj S, López CA, Eeuwema W, Dijkhuizen L, et al. Crystal structure of a 117 kDa glucansucrase fragment provides insight into evolution and product specificity of GH70 enzymes. Proceedings of the National Academy of Sciences. 2010;107(50):21406-11.
2. Case D, Ben-Shalom I, Brozell S, Cerutti D, Cheatham III T, Cruzeiro V, et al. AMBER 18. 2018. San Francisco: University of California[Google Scholar].
3. Arnold K, Bordoli L, Kopp J, Schwede T. The SWISS-MODEL workspace: a web-based environment for protein structure homology modelling. Bioinformatics. 2006;22(2):195-201.
4. Biasini M, Bienert S, Waterhouse A, Arnold K, Studer G, Schmidt T, et al. SWISS-MODEL: modelling protein tertiary and quaternary structure using evolutionary information. Nucleic acids research. 2014;42(W1):W252-W8.
5. Guex N, Peitsch MC, Schwede T. Automated comparative protein structure modeling with SWISS-MODEL and Swiss-PdbViewer: A historical perspective. Electrophoresis. 2009;30(S1):S162-S73.
6. Kiefer F, Arnold K, Künzli M, Bordoli L, Schwede T. The SWISS-MODEL Repository and associated resources. Nucleic acids research. 2008;37(suppl_1):D387-D92.
7. Molina M, Moulis C, Monties N, Pizzut-Serin S, Guieysse D, Morel S, et al. Deciphering an Undecided Enzyme: Investigations of the Structural Determinants Involved in the Linkage Specificity of Alternansucrase. ACS Catalysis. 2019;9(3):2222-37.
8. Gordon JC, Myers JB, Folta T, Shoja V, Heath LS, Onufriev A. H++: a server for estimating p K as and adding missing hydrogens to macromolecules. Nucleic acids research. 2005;33(suppl_2):W368-W71.
9. Jensen MH, Mirza O, Albenne C, Remaud-Simeon M, Monsan P, Gajhede M, et al. Crystal structure of the covalent intermediate of amylosucrase from Neisseria polysaccharea. Biochemistry. 2004;43(11):3104-10.
10. Frisch M, Trucks G, Schlegel H, Scuseria G, Robb M, Cheeseman J, et al. Gaussian 09, Revision C. 01,(2010) Gaussian Inc. Wallingford, CT. 2009.
11. Feig M, Karanicolas J, Brooks III CL. MMTSB Tool Set: enhanced sampling and multiscale modeling methods for applications in structural biology. Journal of Molecular Graphics and Modelling. 2004;22(5):377-95.
12. Nivedha AK, Thieker DF, Makeneni S, Hu H, Woods RJ. Vina-Carb: improving glycosidic angles during carbohydrate docking. Journal of chemical theory and computation. 2016;12(2):892-901.
13. Kirschner KN, Yongye AB, Tschampel SM, González-Outeiriño J, Daniels CR, Foley BL, et al. GLYCAM06: a generalizable biomolecular force field. Carbohydrates. Journal of computational chemistry. 2008;29(4):622-55.
14. Lovell SC, Davis IW, Arendall III WB, De Bakker PI, Word JM, Prisant MG, et al. Structure validation by Cα geometry: ϕ, ψ and Cβ deviation. Proteins: Structure, Function, and Bioinformatics. 2003;50(3):437-50.
15. Götz AW, Williamson MJ, Xu D, Poole D, Le Grand S, Walker RC. Routine microsecond molecular dynamics simulations with AMBER on GPUs. 1. Generalized born. Journal of chemical theory and computation. 2012;8(5):1542-55.
16. Le Grand S, Götz AW, Walker RC. SPFP: Speed without compromise—A mixed precision model for GPU accelerated molecular dynamics simulations. Computer Physics Communications. 2013;184(2):374-80.

17. Salomon-Ferrer R, Götz AW, Poole D, Le Grand S, Walker RC. Routine microsecond molecular dynamics simulations with AMBER on GPUs. 2. Explicit solvent particle mesh Ewald. Journal of chemical theory and computation. 2013;9(9):3878-88.

18. York DM, Darden TA, Pedersen LG. The effect of long-range electrostatic interactions in simulations of macromolecular crystals: A comparison of the Ewald and truncated list methods. The Journal of chemical physics. 1993;99(10):8345-8.

19. Wu X, Brooks BR. Self-guided Langevin dynamics simulation method. Chemical Physics Letters. 2003;381(3-4):512-8.
